# Predictors of powerhouse: a perspective of mitochondrial biomarkers in type 2 diabetes

**DOI:** 10.3389/fendo.2025.1595557

**Published:** 2025-07-28

**Authors:** Mónica Muñoz-Úbeda, Surjya Narayan Dash

**Affiliations:** ^1^ Physical Chemistry Department, Faculty of Chemistry, University Complutense of Madrid, Madrid, Spain; ^2^ Helsinki Institute of Life science, Biocenter 2, Viikinkaari, University of Helsinki, Helsinki, Finland; ^3^ Division of Nephrology, School of Medicine, University of Virginia, Charlottesville, VA, United States

**Keywords:** mitochondrial biomarkers, metabolic syndrome, insulin resistance, type-2 diabetes, mitochondrial dysfunction

## Abstract

Mitochondria play a critical role in maintaining the metabolic balance of the cell. The onset and progression of diabetes have been linked to mitochondrial dysfunction, leading to oxidative stress and dysregulation of metabolic intermediates, ultimately leading to a loss of energy production. Mitochondria play a crucial role in glucose stimulated-insulin secretion in pancreatic β-cells and oxidative phosphorylation in beta cells and skeletal muscles. In type-2 diabetes, impaired oxidative phosphorylation and insulin release is linked to insulin resistance (IR). Given the possible involvement of mitochondrial activity in the pathophysiology of diabetes, it would be highly desirable to investigate possible biomarkers or indicators that may provide details on the onset, severity or progression of the disease. The use of biomarkers is essential both for the diagnosis of mitochondrial diseases and for monitoring their metabolic status. The discovery and characterization of numerous biomarkers that correlate with mitochondrial diseases has led to the development of a number of new biomarkers. Biomarkers associated with human mitochondrial dysfunction are critical for the development of targeted therapies and early diagnosis of diabetes. Based on an investigation of the literature, this perspective outlines the state of knowledge on mitochondrial biomarkers and examines the data supporting their application in the early diagnosis, prognosis, and monitoring of diabetes.

## 1 Type-2 diabetes

In Type 2 diabetes (T2D), the body alters the uses of sugar (glucose) as a source of energy, preventing it from efficiently utilising insulin, which can raise blood sugar levels. T2DM can cause substantial bodily harm over time, particularly to neurones and blood vessels. T2D can be prevented in many cases. Factors that contribute to its onset include being overweight, not getting enough exercise and genetic inheritance. Timely diagnosis is crucial to mitigate the severe consequences of T2D. The best way to detect it early is to see a doctor for regular examinations and blood tests. Symptoms of T2D might be modest and take years to become visible, it includes excessive thirst, increased urination frequency, impaired eyesight, fatigue, and accidental weight loss. Diabetes can eventually cause damage to the blood vessels of the heart, eyes, kidneys, and nerves, increased risk such as heart attack, stroke, kidney failure, and permanent vision loss by damaging the blood vessels in the eyes. Many people with diabetes develop foot problems due to nerve damage and poor blood circulation that can be the major cause of foot ulcers and ultimately lead to amputation (1).

Around 529 million people worldwide were estimated to have diabetes in 2021 by the Global Burden of Diseases (GBD). Injuries, and Risk Factors Study, which evaluated the prevalence and burden of diabetes in 204 countries and territories (2). Globally, approximately one in eleven adults have diabetes mellitus, 90% of whom have T2DM (3). Asia is emerging global epidemic of T2DM rapidly. With a 76·1% (73·1–79·5) of people aged 75–79 having diabetes, Qatar had the highest age-specific prevalence of diabetes in the world. T2D and mitochondrial haplogroups and variants in Arab populations from the Gulf region. Certain mitochondrial DNA variations have been linked to an increased risk of T2D, including the more common mtDNA 16189 T>C and the uncommon mtDNA 3243 A>G. Notably, various populations have distinct connections (4). T2DM leads to a variety of complications that cause serious psychological and financial burdens on patients and society (5). Unhealthy lifestyle habits, such as hypercaloric diets or sedentary lifestyles, are responsible for the increase in metabolic disorders, specifically T2D and/or obesity and associated complications. The importance of knowing why diseases such as diabetes and obesity are increasing so much in our world as an epidemic, not only from a genetic but also from a metabolic point of view, is the central theme of this perspective. High intake of processed food and beverages with high calories, reduced regular physical activities are the major factors contributing to insulin resistance (IR). IR is also associated with physiological stress, oxidative stress, and systemic inflammation. Muscle mitochondrial dysfunction has been found to be closely related to T2D and may lead to IR (6). In addition, the study of mitochondrial biomarkers among different populations is crucial, because ethnic, genetic, age, lifestyle habits such as poor diet and sedentarism and socioeconomic factors can influence biomarker levels (7). To create new therapies to decrease in these diseases, it is crucial to investigate the different mitochondrial biomarkers involved in mitochondrial dysfunction.

Obesity is a disease characterized by the excessive addition of adipose tissue in the body, that is, stored in the form of body fat upsurges to a point that threatens health or life (8). Worldwide the fifth leading death factor for human is obesity. Each year, at least 2.8 million adults die because of obesity. The World Health Organization (WHO) defines obesity when the body mass index (BMI, the ratio of an individual’s weight to height squared) is equal to or greater than 30 kg/m². An abdominal circumference in men equal to or greater than 102 cm and in women equal to or greater than 88 cm is also considered a sign of obesity. Obesity is part of the metabolic syndrome (MetS) and is a known risk factor, that is, it indicates a predisposition to several diseases (although it also causes some of them), particularly cardiovascular disease (CVD), T2DM, sleep apnea, stroke and osteoarthritis, as well as some forms of cancer and dermatological and gastrointestinal conditions. Obesity rates more than doubled globally between 1990 and 2022. It is estimated that 37 million children under the age of five will be overweight by 2022 (9). Obesity is a heterogeneous disease whose exact cause and underlying molecular mechanisms are poorly understood. It alters metabolic status, leading to the development of dyslipidemia, IR, and inflammation; thus, it is a major risk factor for CVD and T2DM. Although obesity causes several problems, the most common of which is T2D, which is often preceded by IR, it has been shown that not all people who gain weight develop IR. An essential endocrine organ that influences metabolism throughout the body is adipose tissue (AT) (8). At some point in the progression of obesity, various stress stimuli may lead to a dysfunctional AT which, in turn, will secrete perturbed signals that may alter the function of adjacent or distant cells and tissues (paracrine and endocrine communications). This altered crosstalk may contribute to inflammation, the development of IR, and decreased mitochondrial oxidative capacity and thus Adenosine Triphosphate (ATP) and Reactive Oxygen Species (ROS) production (10). Obesity and T2D are two serious global health problems that have a major impact on the population and health systems. The fundamental processes linking these two disorders, in particular the function of mitochondria, have attracted considerable interest.

### 1.1 Mitochondrial dysfunction and type 2 diabetes

The mitochondria are thought of as the centre of cellular metabolism because they are the locations for biochemical processes like ATP synthesis, fatty acid synthesis, intracellular ROS generation, oxidative phosphorylation (OXPHOS), which is in the mitochondrial cristae and is composed of the electron transport chain (ETC) complexes and ATP synthase, thermogenesis, and calcium (Ca^2+^) homeostasis. One of the main pathogenic mechanisms of a variety of illnesses and disorders that are typified by faulty mitochondrial function (11). Moreover, mitochondria form a dynamic network of organelles that constantly fuse and divide. The balance between these two antagonistic processes is crucial for cellular function and requires the action of specific proteins. In addition, mitochondria contain their own circular DNA, small circular double-stranded DNA (mtDNA), consisting of 16,569 base pairs and encoding 37 genes—13 for proteins in the ETC, and the rest for rRNAs and tRNAs. Most mitochondrial proteins, however, are encoded by nuclear DNA (nDNA), including key regulators like mitochondrial transcription factor A (TFAM), which controls mtDNA transcription and replication (12). Mitochondria have four compartments: a) outer membrane which is permeable to ions and small molecules; b) intermembrane space where proton accumulation creates an electrochemical gradient; c) inner membrane where the ETC complexes are located and where the ATP is produced; and d) the matrix, where is produced the pyruvate and fatty acid oxidation. The inner membrane’s cristae increase surface area, boosting ATP production. Mitochondria are dynamic and adapt through biogenesis, fusion and fission. Biogenesis is mainly controlled by peroxisome proliferator-activated receptor-gamma coactivator-1alpha (PGC-1α) and peroxisome proliferator-activated receptor-gamma coactivator-1beta (PGC-1β), which activate oxidative phosphorylation genes and TFAM via NRF-1. PGC-1α also regulates thermogenesis, fatty acid breakdown, and glucose uptake. Autophagy and other ongoing remodelling are essential for mitochondrial function and repair. These features make mitochondria central to both energy metabolism and cell fate decisions. Mitochondria are essential for ATP production via oxidative phosphorylation, ROS generation, and apoptosis regulation, all of which are important in diabetes. In TCA cycle and β-oxidation, glucose and fatty acids are metabolized in mitochondria producing NADH and FADH_2_. These transfer electrons into the ETC, resulting in a proton gradient that drives ATP production. Metabolic regulation relies heavily on mitochondria, which play a crucial role in energy homeostasis by metabolising foods and creating ATP and heat. Imbalanced energy intake and expenditure causes mitochondrial dysfunction, which is characterised by a lower ratio of energy generation to respiration. During metabolic diseases, different changes in mitochondrial function, dynamics, and biogenesis result from the genetic and epigenetic regulation of mitochondria, the primary metabolic platform in mammalian cells (13).

Diabetes is known to be exacerbated by mitochondrial dysfunction for a variety of reasons. Over the last few years, research has shown a close relationship between mitochondrial function and diabetes in the context of pancreatic β-cell malfunction, IR, obesity, and vascular problems. It has been demonstrated that mitochondrial function is declining, which could be a factor in the onset of heart disease and T2D (14). Crucially, it has been demonstrated that IR in skeletal muscle, as well as in other tissues such the liver, fat, heart, arteries, and pancreas, is linked to mitochondrial malfunction. Reduced energy production, elevated formation of reactive oxygen species (ROS), and compromised insulin signalling are the results of age-related declines in mitochondrial activity. Therefore, age-related IR and T2D may be linked to a shared pathophysiological etiology for many chronic diseases including IR (15, 16).

Another contributing factor to diabetes is the possibility of electron leakage and the formation of ROS like superoxide when the ETC is compromised by an excess of nutrients or a limited capacity. These ROS have the ability to harm lipids, mitochondrial proteins, and mtDNA, which is particularly susceptible because of inadequate repair systems. Diabetes may be aggravated by this damage, which can also increase ROS production. When under stress, mitochondria also release proteins like cytochrome c, which causes apoptosis. Inactivity and overeating can cause cells to produce more ROS and undergo apoptosis, which accelerates the course of disease. Moreover, in the early 1990s, a specific mtDNA mutation (A3243G) linked to maternally inherited diabetes was identified. This mutation, affecting the leucyl-tRNA^UUR gene, is found in about 1% of diabetes patients, with higher prevalence (up to 10%) in those with atypical type 1 diabetes. It can present as maternally inherited diabetes and deafness (MIDD) or MELAS syndrome (17, 18). The degree of heteroplasmy is associated with earlier onset and higher HbA1c levels, with β-cell dysfunction being more prominent than IR. The A3243G mutation lowers ATP synthesis and oxygen consumption. Furthermore, mitochondrial function is also hampered by abnormalities in nuclear genes. For instance, mutations in HNF-4α and HNF-1α reduce ATP production and insulin secretion by affecting pyruvate oxidation and mitochondrial coupling. Mutations in IPF-1 further impair mitochondrial function by downregulating TFAM in β-cells. These findings show that both mtDNA and nDNA mutations contribute to mitochondrial dysfunction and the development of diabetes (19).

Conversely, hyperglycaemia and hyperlipidaemia can arise as a result of another factor that affects diabetes at the level of mitochondrial dynamics and biogenesis (20, 21). Lessened mitochondrial fusion and increased mitochondrial fission can result from glutalipotoxicity (22). Secreted frizzled related protein (SFRP) family members were recently found to be involved in the pathogenesis of a variety of metabolic diseases, which has led to extensive interest in SFRPs (23). Previous reports highlighted the importance of SFRPs in lipid metabolism, obesity, T2DM and CVD and signalling pathways and metabolic disease impacts (24). In addition to summarizing the pathologies and potential molecular mechanisms associated with SFRPs, we further suggest the potential future use of SFRPs as disease biomarker therapeutic targets (25).

A possible solution to improve certain influencing factors in diabetes is the use of metformin. Metformin, and other key antidiabetic medications, as well as insulin-sensitizing adipokines (like adiponectin), are likely to target a phylogenetically conserved serine/threonine protein kinase. Metformin helps control metabolism and energy balance by activating AMPK (AMP-activated protein kinase). Targeting the AMPK pathway for the treatment of T2D is strongly supported by the evidence that has been gathered over the last few years, which shows that AMPK functions as an integrator of regulatory signals monitoring systemic and cellular energy status (26). Therefore, modulating AMPK signalling to enhance mitochondrial activity could be an immense way to treat T2D. A fuel-sensing enzyme called AMPK is found in every mammalian cell. It is triggered during exercise, raises the human AMP/ATP ratio. Fatty-acid oxidation and glucose transport are two mechanisms that AMPK initiates, boosts ATP generation. Further, it might especially promote heart muscle glycolysis. Furthermore, AMPK activation in peripheral tissues appears to mitigate a number of the MetS’s cellular abnormalities, such as inflammation, ectopic lipid deposition, and IR. On the other hand, these anomalies can be exacerbated by its dysregulation, which is characterised by reduced activity or impaired activation (27). AMPK activation produces insulin-sensitizing effects, it appears to be a desirable and viable target for the pharmaceutical treatment of T2D. On a low cellular energy level, the energy-sensing enzyme AMPK is activated and signals to increase glucose absorption and decrease hepatic glucose synthesis. AMPK can be directly or indirectly activated by a wide range of pharmacological drugs, natural substances, and hormones; some of these are now used to treat T2D, including metformin and thiazolidinediones. To activate AMPK, 5-aminoimidazole-4-carboxamide riboside (AICAR) was the first substance discovered. AICAR’s short half-life and poor bioavailability make it unlikely to be employed in the treatment of MetS or T2D in humans. Presently physicians recommend metformin, an insulin-sensitizing medication, as the first-line oral therapy for T2D (28). Troglitazone, pioglitazone, and rosiglitazone are members of the class of insulin-sensitizing medications known as TZDs. They are believed to have some antidiabetic effects through AMPK activation, even though their main target is the nuclear hormone receptor peroxisome proliferator-activated receptor-γ (PPARγ) (29). However, TZDs’ primary disadvantages is they raise the risk of bladder cancer and cause weight gain (30). Following food consumption, intestinal L-cells release the incretin glucagon-like peptide-1 (GLP-1). According to recent research, the AMPK pathway can be activated by endogenous GLP-1 (31). The initial compound found to be an AMPK direct activator was A-769662 (32). Excessive activation of AMPK can have unintended repercussions, even though there is growing evidence, both *in vitro* and *in vivo*, that it positively impacts many physiological systems that are dysregulated in metabolic disorders. For instance, AMPK prevents the synthesis of proteins, which may be detrimental, especially to elderly people (33). In addition to being crucial for comprehending how exercise affects muscle mitochondria, AMPK’s impact on mitochondrial biogenesis also has therapeutic implications in several clinical conditions. Data suggests that AMPK specifically regulates a number of elements of mitochondrial biology and homeostasis includes regulating the architecture of the mitochondrial network, controlling the number of mitochondria by inducing mitochondrial biogenesis, and quality of mitochondria by controlling autophagy and mitophagy.

## 2 Mitochondrial biomarkers and type 2 diabetes

**Figure 1:**
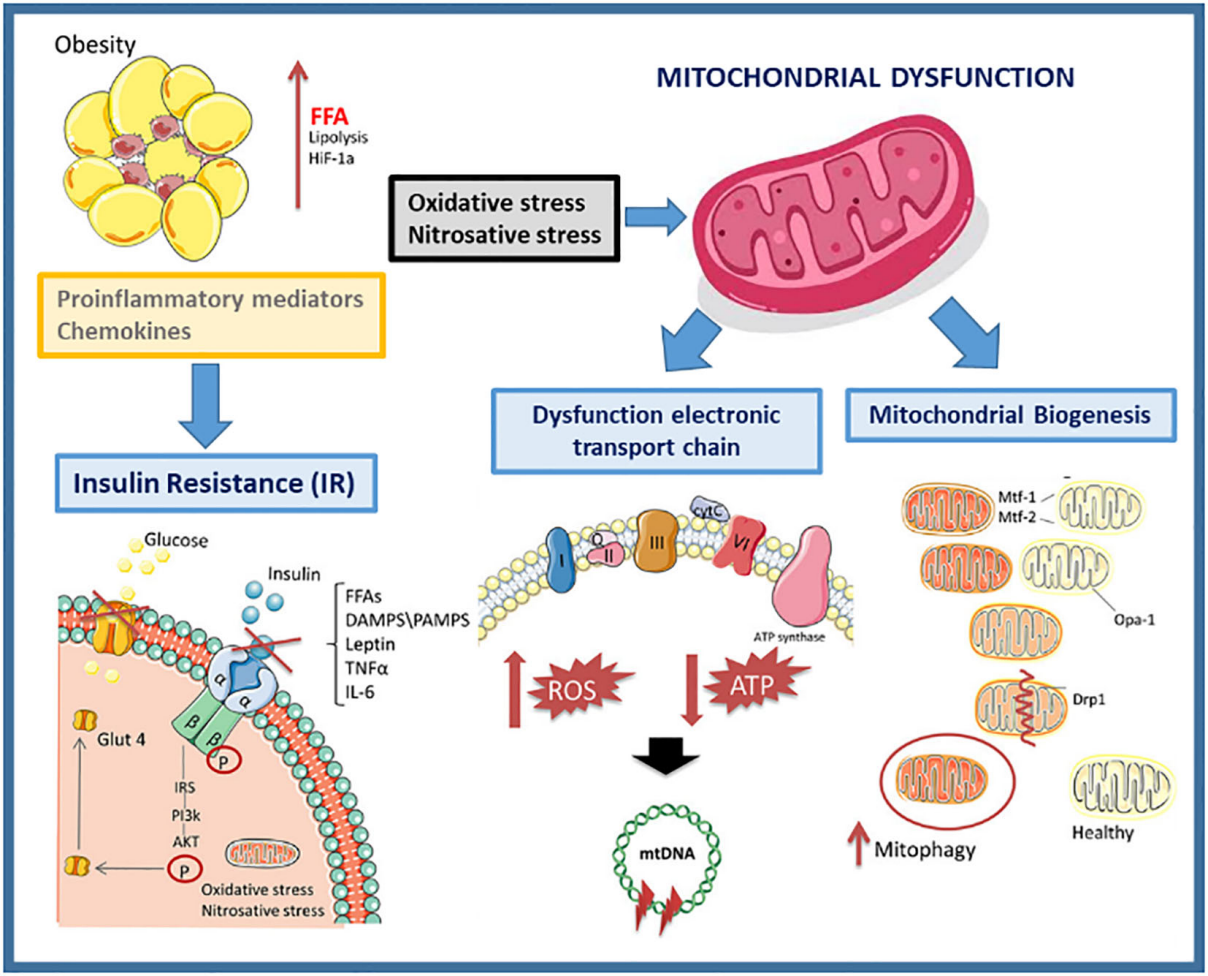
Mitochondrial dysfunction in obesity.

Understanding the role of mitochondria is also crucial for conducting a more thorough investigation into the prevention or manifestation of diabetes and other related disorders. When an overt MetS is present in T2D and/or obesity patients, an inflammatory process occurs that can lead to an increase in the production of ROS and oxidative stress (**Figure 1**).

Mitochondrial biomarkers are the oxidative stress caused by an imbalance between the production of ROS and the ability of a biological system to rapidly neutralize the reactive intermediates or repair the resulting damage. Excessive nutrient supply (as is often the case with excess weight) can overload the Krebs cycle and the mitochondrial respiratory chain, leading to mitochondrial dysfunction with an increased ROS formation, exacerbating the inflammatory process in obesity. To uncouple the ETC and lower ATP generation, mitochondria divide when there are too many nutrients present (31).

Other crucial mitochondrial biomarkers in T2DM are related with the mitochondrial membrane proteins involved in the fusion and fission processes, necessary for a normal cellular function. The mitochondrial membrane proteins Mitofusin 1 (Mfn1) and Mitofusin 2 (Mfn2) are responsible for the fusion of the outer membrane (OMM) of adjacent mitochondria, while the membrane protein OPA1 is responsible for the fusion of inner mitochondrial membranes (IMM)). Mutations in Mfn1, Mfn2 or Opa1 trigger the imbalance of mitochondrial fusion and produce different severe mitochondrial dysfunctions and mitochondrial diseases (MD). A characteristic phenotype of cells that have a defect in these proteins is that they present a highly fragmented mitochondrial network. Several studies have documented the essential role of mitochondria in adipocyte differentiation, lipid metabolism, and thermogenesis. The balance of mitochondrial fusion and fission, which alters morphology and abundance, is important for mitochondrial function and the maintenance of a healthy mitochondrial network (34). It has been studied that the modulation of mitochondrial fusion and fission processes can influence adipose tissue. Intra-abdominal or visceral adiposity is the most common cause of IR and has been identified as a powerful etiopathogenic factor for the development of T2DM and accelerated atherogenesis. Intra-abdominal fat has metabolic and anatomical characteristics that favour IR, pro-inflammatory characteristics and pro-coagulant states, which explains its proatherogenic function. Tissue-specific changes in mitochondrial function accompany obesity and IR in animals, suggesting that the metabolic flexibility and function of each organ or cell type affect mitochondrial adaptations to an excessive food supply and inactivity.

**Figure 2:**
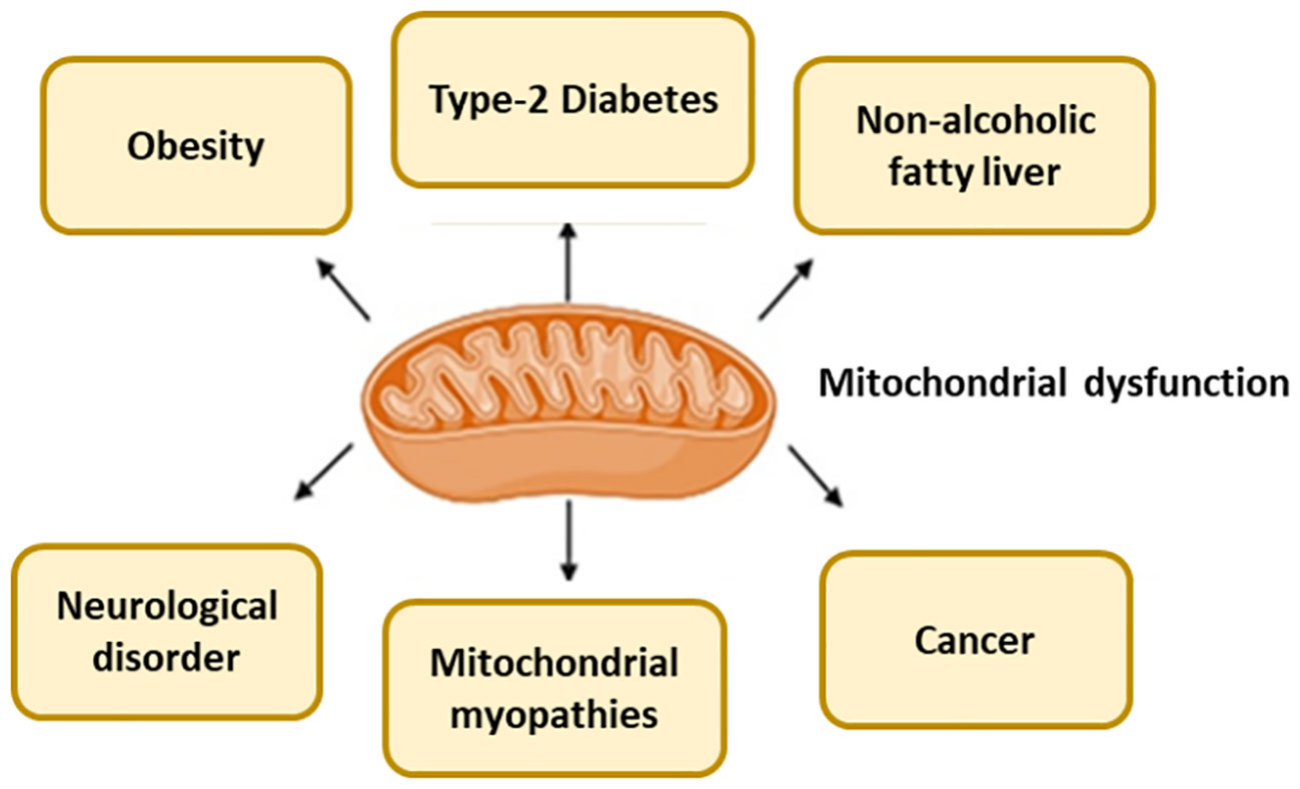
Mitochondria dysfunction cause several diseases.

**Figure 3:**
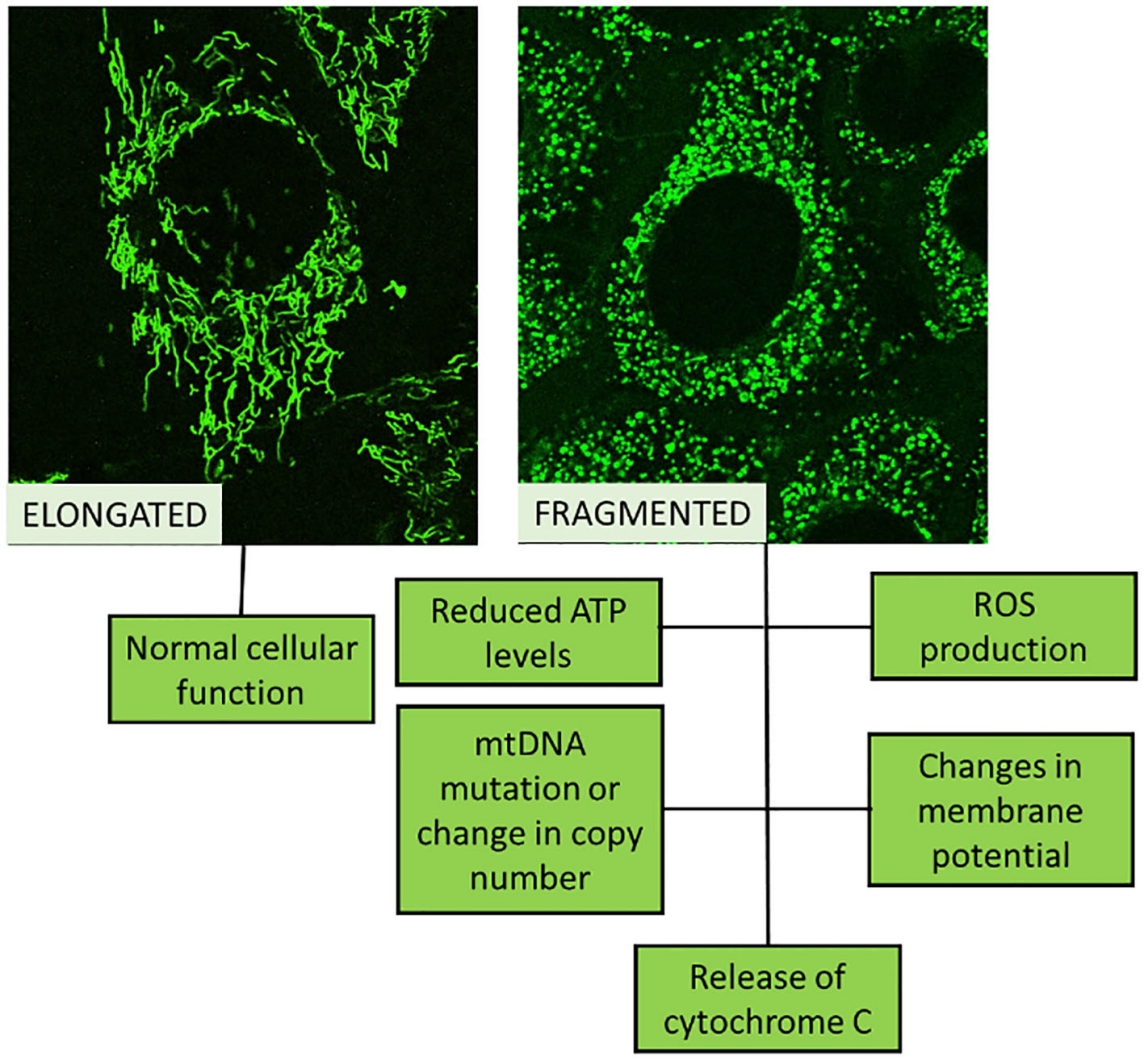
Schematic representation of normal cellular function (healthy mitochondria looks elongated) compared to de-regulated cellular function (fragmented mitochondria).

For evaluating mitochondrial health, function, or disease, mitochondrial biomarkers are used (**Table 1**). A number of diseases, including MetS, neurological conditions, and some cancers, are associated with abnormalities in mitochondrial activity (**Figure 2**), which also depends on whether the mitochondria are undamaged or fragmented (**Figure 3**). Measuring common mitochondrial biomarkers like ATP levels, ROS, mtDNA, cytochrome c, membrane potential and mitochondrial biogenesis markers are key for detecting human diseases related to mitochondria. MetS are caused by differences in the amounts of lactate and pyruvate or the NAD+/NADH ratio, which affect metabolism. By monitoring these biomarkers, researchers and physicians can gain insight into the fundamental causes of mitochondrial dysfunctions and create specialised treatments for illnesses related to mitochondria.

**Table 1:** Potential mitochondrial biomarkers that are associated with the onset of diabetes.

Methods to detect mitochondria health	Biomarkers linked to mitochondrial dysfunction
Respiratory Chain Enzyme Assays	Lactate, Pyruvate, Lactate: Pyruvate ratio, creatine kinase, creatine, amino acids, NAD+/NADH ratio
Mitochondrial DNA Analysis (genetic/epigenetic modifications)	MiR-27b-3p, POLG (DNA Polimerase Gamma), DNM1L gen (encodes Drp1), MIPEPc.304>T, m.10225T>G, 3243A>G/MT-TL1, ccf mtDNA, miR-378
Omic profiling	NDU5S3 (NADH dehydrogenase [ubiquinone] iron-sulfur protein 3), COX2 (cyclooxygenase 2), CALR (calreticulin), SORT (Sortilin 1), COX6B1 (cytochrome oxidase c subunit 6B1 enzyme), COX6C (cytochrome c oxidase enzyme subunit 6C), TXN2 (Thioredoxin 2), SOD2 (superoxide dismutase 2), TFAM (Transcription Factor A Mitochondrial), SDHC (Succinate Dehydrogenase Complex Subunit C), CS (citrate synthase), MT-CO1 (Mitochondrially Encoded Cytochrome C Oxidase I), MT-CO2 (Mitochondrially Encoded Cytochrome C Oxidase II), TOMM70A (Translocase Of Outer Mitochondrial Membrane 70), TOMM20 (Translocase Of Outer Mitochondrial Membrane 20), RAB1A (Member RAS Oncogene Family)
Mitochondrial Dynamic Analysis	Mfn1 (Mitofusin 1), Mfn2 (Mitofusin 2), Opa1 (Optic Atrophy 1), Drp1 (Dynamin related protein 1), mdivi-1 (Mitochondrial division inhibitor-1, a Drp1 inhibitor), Fis1 (Fission 1 protein)
Mitophagy Dysfunction Analysis	PINK1 (PTEN-induced kinase 1), Parkin
Oxidative stress Analysis	ROS levels, lipid peroxidation, protein carbonylation, DNA damage
Mitochondrial Function Analysis	TAC (Total Antioxidant Capacity), GR (Mitochondrial Glucocorticoid Receptor), NOS (Nitric Oxide Synthase), CARB (Carbohydrates), H_2_O_2_, F_0_/F_1_-ATPase (Adenosine Tryphosphate synthase), ATP production, mitochondrial membrane potential (ψ_mit_), PHB (Prohibitin), UCP2 (Uncoupling Protein 2)
Mitochondrial Dysfunction Analysis	Acyl Carnitine (AC), Citrate synthase (CS), cytochrome C (CC)
Mitochondrial Peptide Analysis (Metabolomics)	Humanin (HN), MOTSc (Mitochondrial open reading frame of the 12S rRNA type-C), CRP (C-Reactive Protein), IL-6 (Interleukin-6), IGF-1 (Insulin-like Growth Factor 1), MCP1 (Monocyte Chemoattractant Protein-1), IL-10 (Interleukin-10)
Mitochondrial Biogenesis Analysis	PCG-1α (Peroxisome Proliferator-Activated Receptor-gamma Coactivator 1-alpha), SIRT1 (Sirtuin Protein 1), GLP-1 (Glucagon-Like Peptide 1), AMPK (AMP-activated protein kinase), GLUT4 (Glucose Transport 4), SFRP (Secreted Frizzled-Related Protein), AICAR (5-aminoimidazole – 4 – carboxamide – 1 - beta – D -ribofuranoside)

Research indicates that damaged mitochondria impair oxidation and the synthesis of ATP, which interferes with insulin signalling (**Figure 3**). As crucial parts of the mitochondrial apparatus, cytochrome c (CC), acylcarnitine (AC), and citrate synthase (CS) can be employed as trustworthy biomarkers of mitochondrial malfunction (**Table 1**). Researchers looked at the relationship between IR and mitochondrial biomarkers (AC, CS, and CC) and if people with T2DM had different amounts of these biomarkers. Overall, AC, CC, and CS measurements of mitochondrial biomarkers showed alterations in T2DM patients that were directly associated with IR (35).

The activity and quality of mitochondria depends on mitochondrial content, biogenesis, renewal and dynamic remodelling, including fusion, fission, movement and mitophagy within the cell. A decrease in the number of copies of mtDNA has been observed in skeletal muscle, adipose tissue and peripheral blood of obese individuals and individuals with T2DM (36, 37). Variants in mtDNA disrupt energy metabolism, oxidative stress response, insulin production, and mitochondrial function, all of which have a significant impact on T2DM. Maternally inherited diabetes and deafness (MIDD), in which hearing loss and early-onset diabetes are important features, is linked to the m.3243 A > G variation (17). T2D susceptibility is increased by ND4 and tRNA Ala genes, which are involved in other forms of mtDNA variations. Gaining knowledge of these variations will serve as the foundation for creating tailored treatments that enhance metabolic health and mitochondrial function (38).

In diabetic patients, an excess of mtDNA passed by the kidney may result in chronic renal inflammation. Reduced plasma mtDNA content and an increased urine mtDNA/creatinine ratio may be early signs of diabetic nephropathy (39). Furthermore, studies conducted in the last four decades have determined that the development of obesity and T2DM are mechanistically linked to skeletal muscle mitochondrial dysfunction (40, 41). Mitochondrial dysfunction in skeletal muscle contributes to several aspects of the MetS, likely due to the pathological accumulation of intracellular lipids and lipid intermediates that inhibit metabolic signalling insulin (40, 42, 43).

On the other hand, mitochondrial activity relies on several proteins, mostly encoded by nuclear DNA in humans, but proteins involved in super complexes of the ETC are encoded by the mitochondrial genome, mtDNA, play a fundamental role in its function. Inherited mutations in mtDNA have been linked to several genetic disorders including T2DM, deafness, obesity, and Leber hereditary optic neuropathy. In other studies, it has also been observed that there is a reduction in mtDNA content in a group with obesity compared to a group that does not have obesity, with mtDNA content being positively correlated with BMI. The process by which excess energy substrates cause IR and inflammatory cytokine profile is what drives mtDNA CN (copy number) alteration in metabolic disorders. This process leads to oxidative stress (increased production of ROS), mitochondrial fragmentation, accumulation of mtDNA damage, and cellular senescence (44).

The majority of evidence is in favour of the idea that optimal glucose-induced insulin production requires proper mitochondrial function to be maintained. In order to comprehend the mechanisms that mitochondria employ to deregulate their biogenesis and function, resulting in obesity, IR, T2D, deafness, ageing, or CVD, among other conditions, it is important to investigate not only the metabolomic, lipidomic, inflammatory, and epigenetic profiles, but also the mtDNA content, mitochondrial dynamics and morphology, mitophagy (PINK1 and Parkin proteins, among others), biogenesis, and functions. Examining potential improvements in mtDNA content, mitophagy, morphology, dynamics, biogenesis, and mitochondrial function as well as different indices involved in glycaemic metabolism and inflammatory biomarkers related to obesity and T2D or to other diseases, it is possible to find a potential cure or improve the quality of life of patients.

## 3 Clinical implications of mitochondrial biomarkers

Research indicates that impaired mitochondrial function can reduce β-oxidation and hinder the production of ATP. In adult glucose metabolism, disruptions in mitochondrial function have been associated with IR and diabetes in humans (45, 46). Mitochondrial dysfunction can be assessed using blood biomarkers or enzymes, including AC, CC, and CS. AC is essential for transporting long-chain fatty acids across the IMM, facilitating oxidation and ATP generation. Carnitine helps eliminate acyl residues and regulates coenzyme A, which is crucial for maintaining mitochondrial membrane integrity (47, 48). CS is a key enzyme that catalyses the conversion of acetyl-CoA and oxaloacetate into citrate and CoA in an irreversible reaction. As a rate-limiting step in the TCA cycle, CS is commonly used in research to measure mitochondrial content, activity, and function (49, 50). CC, located in the IMM, plays a vital role in electron transport by transferring electrons from complex III to complex IV, a fundamental process for mitochondrial energy metabolism. In cases of mitochondrial dysfunction, CC is released as a marker of cellular distress (51). Their study aimed to evaluate the levels of AC, CS, and CC as biomarkers of mitochondrial dysfunction and examine their relationship with IR in individuals with T2DM without the need for a biopsy or mtDNA analysis.

Mitochondrial dysfunction is increasingly recognized as a key contributor to the development of diabetes. Mitochondria which serve as the central hub for metabolic pathways essential for the function of insulin-producing β-cells as well as insulin-responsive tissues such as the liver, muscle, and adipose tissue (52). In pancreatic β-cells, mitochondria play a crucial role in insulin secretion. During glucose metabolism, ATP is produced, leading to the closure of ATP-sensitive potassium channels, cell depolarization, and subsequent insulin release. This process, known as glucose-stimulated insulin secretion, is heavily dependent on mitochondrial function. Therefore, abnormalities in mitochondrial glucose metabolism can affect β-cell activity and trigger the development of T2D (53). Furthermore, mitochondrial dysfunction in insulin-sensitive tissues can contribute to IR, a defining characteristic of T2D. Reduced ATP production and increased ROS generation can interfere with insulin signaling, ultimately leading to IR (42). Furthermore, mtDNA mutations and the resultant inefficiencies in oxidative phosphorylation have been related to diabetes. Research indicates that mtDNA mutations can cause β-cell malfunction and IR, potentially contributing to diabetes development (54).

The function of mitochondria in *in vitro* cells and animal studies has been investigated in lab settings. The significance and use of mitochondrial biomarkers in therapeutic settings, however, are less well understood. OXPHOS insufficiency has been associated with altered protein expression of the complex components, which leads to apoptosis and ROS generation, which damages cells. In a Finish cohort of monozygotic twin discordant, Heinonen et al., 2015 (55) found that global expressional downregulation of mitochondrial oxidative pathways with concomitant downregulation of mtDNA amount, mtDNA-dependent translation system, and protein levels of the OXPHOS machinery in the obese were examined in obese and lean co-twins. Takamura et al., 2008 (56) found that obesity may alter the pathophysiology of T2D patients by upregulating genes involved in OXPHOS in conjunction with IR indicators, as well as the expression of genes involved in hepatic gluconeogenesis and ROS production. Bordoni et al., 2019 (57) collected mtDNA from buccal of a young Caucasian population studied potential correlation between mtDNA copy number and methylation with body composition. In females, mtDNA copy number was found to be negatively correlated with BMI. On the other hand, the greater urine mtDNAcn detected in obese individuals relative to lean participants decreased after bariatric surgery, implying that lower mtDNAcn is related with better metabolic health (58). Additionally, leukocyte mtDNAcn was significantly lower in obese individuals and inversely correlated with BMI. Therefore, it is conceivable that alterations in mitochondrial dynamics and cellular function caused by mtDNAcn and mtDNA epigenetic modifications could both increase the risk of obesity (59). In the context of metabolic disease, the effects of mtDNA variations, whether homoplasmic or heteroplasmic, can be catastrophic. T2DM has been linked to a long list of single nucleotide polymorphisms and mtDNA mutations. To ascertain whether a particular haplogroup is linked to a higher or lower risk of T2D, some groups have divided their study populations into mitochondrial haplogroups based on mtDNA polymorphisms (60, 61). Because mitochondrial failure is close to ROS and lacks histone protection, it damages mtDNA, which causes systemic inflammation and renal impairment. Furthermore, dysregulated mitochondrial dynamics due to compromised mitophagy exacerbate MD and DN (62).

The pathophysiology of diabetic cardiomyopathy is also significantly influenced by oxidative stress and inflammation, which encourage cardiac damage and remodelling. Pro-inflammatory cytokines such IL-1β and IL-6, as well as increased ROS generation, are linked to cardiac dysfunction, inflammation, and oxidative damage (63). Increased fatty acid oxidation caused by MD causes mitochondrial uncoupling in diabetic cardiomyopathy, which is marked by increased proton leakage and decreased ATP regeneration (64). IMM proteins prohibitin (PHB) and uncoupling protein-2 (UCP2) have been shown to improve both mtROS and mitochondrial membrane potential (Δψm). Circulating levels of PHB and UCP2 in people with and without T2D may serve as biomarker surrogates for vascular health. T2DM patients reported lower circulating UCP2 levels, which is linked to endothelial-dependent conduit channel vasodilation. UCP2 needs further investigation because it may be a biomarker surrogate for overall vascular health in T2DM patients (65). Mitokines released in response to this mitochondrial stress can influence the development and progression of diabetes. Mitokines, a specific class of cytokines released by mitochondria in response to stress, play a significant role in metabolic regulation and have implications for conditions like diabetes. For example, they may modulate insulin sensitivity, glucose uptake by cells, and overall energy metabolism. Numerous mitokines were shown to be engrossed in diabetes (66). A well-researched mitokine Fibroblast Growth Factor 21 (FGF21) known for its valuable effects on glucose and lipid metabolism. It enhances insulin sensitivity and has been linked in the regulation of body weight and energy expenditure. FGF21′s protective effects against metabolic stress make it a promising target for diabetes therapy. In addition, GDF15 levels increase in response to mitochondrial dysfunction. It has been linked to the regulation of body weight and appetite. Elevated GDF15 levels have been observed in individuals with diabetes, suggesting its role in the disease’s metabolic disturbances (67).

## 4 Recent advances in diabetic biomarker research

Understanding the relationship between mitochondria and diabetes has led to the development of innovative therapeutic approaches. Treatments targeting mitochondria aim to maintain mitochondrial health, enhance oxidative capacity, and correct metabolic imbalances in diabetes. Antioxidants specifically designed for mitochondria, such as MitoQ (68) and SkQ1 (69), help counteract oxidative stress and restore mitochondrial function. These antioxidant-based therapies show promise in reducing oxidative damage and improving mitochondrial efficiency in diabetes management. MitoQ, SkQ1, and SS peptides have demonstrated effectiveness in experimental diabetes models, highlighting their potential as therapeutic agents. Ongoing research in this field will offer further insights into the benefits of these compounds and their role in treating diabetes. By reducing oxidative stress, mitochondria-targeted antioxidants contribute to better mitochondrial and cellular function. MitoQ has been shown to improve mitochondrial function and reduce oxidative stress in experimental diabetic models (68). Although MitoQ successfully lowers oxidative damage brought on by exercise, there is no proof that either acute or long-term MitoQ treatment improves aerobic exercise performance (70, 71). Shill et al., examined the potential effects of mitochondria-specific antioxidant (MitoQ) supplementation on young, healthy men’s maximal oxygen uptake, muscle mitochondrial capacity, and responsiveness to three weeks of endurance exercise training in circulating angiogenic cells (CACs). CACs are an exercise-inducible subset of white blood cells that maintain vascular integrity, in which, numerous CAC types are increased by endurance exercise training, that is unaffected by MitoQ supplementation. Furthermore, skeletal muscle and whole-body aerobic responses to exercise training are unaffected by MitoQ. According to these findings, endurance training is neither enhanced nor diminished by MitoQ supplementation. It is important to see the future studies whether using MitoQ supplements at the same time as exercise training changes the adaptations brought on by exercise alone and by varying the dosage.

The genetic factors contributing to mitochondrial dysfunction in diabetes have also become a key focus of scientific inquiry. Mutations in mtDNA and nuclear DNA responsible for encoding mitochondrial proteins can disrupt mitochondrial function, increasing the risk of diabetes (72, 73). Certain mtDNA mutations, such as A3243G, are linked to mitochondrial diabetes and maternally inherited diabetes and deafness (MIDD) (70). Additionally, epigenetic modifications—such as DNA methylation, histone changes, and non-coding RNAs—can influence mitochondrial gene expression, impacting mitochondrial function and contributing to diabetes development (74). The human mitochondrial genome encodes 13 essential proteins, all of which play a crucial role in the ETC. Due to its limited repair mechanisms, mtDNA is highly susceptible to mutations, which can disrupt oxidative phosphorylation, leading to reduced ATP production and increased ROS generation. The A3243G mutation, in particular, has been strongly associated with mitochondrial diabetes, frequently accompanied by deafness (75). While mtDNA contributes to mitochondrial protein synthesis, most mitochondrial proteins are encoded by nuclear DNA. Mutations in these nuclear genes can result in various mitochondrial disorders, some of which have been linked to diabetes (76–78). Epigenetic modifications, including DNA methylation and histone modifications, play a significant role in regulating mitochondrial function and influencing diabetes progression. DNA methylation, which encompasses the addition of methyl groups to DNA molecules, modifies gene expression patterns. In diabetes, abnormal DNA methylation can impact the expression of nuclear-encoded mitochondrial genes, leading to mitochondrial dysfunction. For example, hypermethylation of the PGC-1α promoter—an essential regulator of mitochondrial biogenesis—has been observed in individuals with IR and T2D (79). This hypermethylation results in decreased gene expression and impaired mitochondrial activity, potentially contributing to metabolic disorders in diabetes. Histone modifications, which involve chemical changes to the proteins around which DNA is wrapped, also influence mitochondrial function and diabetes progression. Reduced expression of SIRT1, a histone deacetylase involved in gene regulation, has been observed in the skeletal muscle of T2D patients (80). This decrease in SIRT1 expression is linked to impaired mitochondrial function due to altered histone acetylation, which can affect gene activity and mitochondrial performance. Changes in histone modifications may disrupt the normal regulation of mitochondrial genes, further contributing to mitochondrial dysfunction in diabetes. Besides, non-coding RNAs, particularly microRNAs (miRNAs), have emerged as key regulators of mitochondrial function and are implicated in diabetes. MiRNAs play a key role in regulating gene expression and influencing cellular functions like cell death and proliferation. Their dysregulation can impair insulin signaling, contributing to the development of T2DM. MiRNAs can influence mitochondrial activity by directly targeting mitochondrial genes or by regulating nuclear-encoded mitochondrial gene expression. For example, miR-378, which has been found to be dysregulated in diabetes, targets both the mitochondrial gene COX1 and nuclear-encoded mitochondrial genes, suggesting its role in mitochondrial dysfunction in diabetes (81). Specific miRNAs—such as miR-375, miR-126, Let-7, and others—have been frequently associated with diabetes and its complications, suggesting their diagnostic relevance (82). ROS generated by mitochondria significantly contribute to cellular oxidative stress, which is a major factor in the development of diabetes.

Mitochondrial biogenesis, the process of forming new mitochondria, has been explored as a potential therapeutic approach for improving mitochondrial function and metabolic control in diabetes. Activators of PGC-1α, such as resveratrol, have been shown to enhance mitochondrial energy production and improve metabolic function (83, 84). PGC-1α is a critical regulator of mitochondrial biogenesis, and compounds that increase its expression or activity may hold therapeutic potential. Resveratrol, a natural compound found in grapes and berries, has demonstrated beneficial effects in experimental diabetes models by stimulating PGC-1α through SIRT1 activation (85). Its effects include promoting mitochondrial biogenesis, reducing oxidative stress, and improving insulin sensitivity. In the context of IR, AICA ribonucleotide (AICAR) has shown potential in reducing peripheral IR. AICAR has been reported to help alleviate IR (86), with studies in animal models consistently showing improvements in metabolic function following AICAR administration (87–89).

Targeting mitochondrial dynamics, which involves regulating the balance between mitochondrial fusion and fission, is another promising therapeutic strategy for diabetes (90, 91). Disruptions in these processes can lead to mitochondrial dysfunction, but restoring equilibrium may improve mitochondrial health. For example, mdivi-1, a pharmacological inhibitor of the fission protein Drp1, has shown potential in experimental models by improving mitochondrial function and insulin sensitivity (92).

Given the growing evidence supporting mitochondrial-targeted therapies, these strategies hold promise for diabetes treatment. However, further preclinical and clinical studies are necessary to fully evaluate their effectiveness and safety.

The main mitochondrial biomarkers studied that are related with the onset of T2DM and obesity are collected in **Table 1**.

## 5 Conclusion and future perspective

To improve our understanding and use of mitochondrial biomarkers in clinical settings, it will be crucial to bolster evidence from a variety of groups while appropriately accounting for social and environmental confounders. Before obesity develops, mitochondrial profiles could be helpful for predicting long-term metabolic health. The study of different mitochondrial biomarkers (involved in mitochondrial dynamics, mitophagy, oxidative phosphorylation and biogenesis) and possible mutations in the mitochondrial genome (mtDNA) in the prevention/appearance of diabetes is of great importance. WHO involved in establishing guidelines for mtDNA analysis and sequencing, especially when it comes to mitochondrial disorders. This is essential for precise diagnosis and comprehension of illnesses associated with mitochondrial dysfunction. Improving mitochondrial function may help prevent or treat diabetes. Calorie restriction is effective in reversing IR and enhancing mitochondrial activity, largely through SIRT1, a key regulator. SIRT1 promotes insulin secretion, protects β-cells from oxidative stress, and activates PGC-1α to boost mitochondrial biogenesis. Small molecules that activate SIRT1 can mimic calorie restriction, improving insulin sensitivity and mitochondrial function (93, 94). Type 2 diabetics exhibited alterations in mitochondrial markers, these biomarker changes demonstrated a direct connection with IR, however, confirmation from a larger sample group is required to determine the possible significance of these findings.

Antioxidants can be employed as a prophylactic measure or as a therapeutic treatment for metabolic diseases. Biomarkers and molecular targets may aid in the development of novel approaches for the diagnosis, prevention, and treatment of inflammatory and metabolic disorders. We propose that maintaining an appropriate level of glucose-induced insulin secretion depends on proper mitochondrial function that also determines the higher or lower risk of T2DM. We offer these subjects as possible directions for further research in the context of illness and health. To precisely identify and describe the alterations at the mitochondrial level as well as the therapeutic strategies, extensive and intricate research is required.

We also suggest that additional new approaches to enhancing these mitochondrial indicators through a particular diet, exercise regimen, or other means including an investigation regarding the potential correlation between the emergence of disorders like obesity, diabetes, ageing, hypertension, and dyslipidaemia and mitochondria, their genome, and mitochondrial dynamics. It can help increase cellular respiration by generating more proteins involved in mitochondrial dynamics and oxidative phosphorylation, reducing oxidative stress and increasing mitochondrial O_2_ consumption for proper functioning, thus reducing all possible cardio-metabolic risks derived. Therefore, a more exhaustive study of mitochondrial dynamics and biogenesis throughout the life cycle from childhood to old age, and in populations with WHO and SOM, are crucial for a greater understanding of the involvement of mitochondria in appearance of obesity, diabetes, hypertension, dyslipidaemia and its cardiometabolic risks. To maximise the efficacy and safety of mitochondrial-targeted therapeutics, more accurate and focused approaches as well as improvements in drug delivery technologies would be essential.

## Data Availability

The original contributions presented in the study are included in the article/supplementary material. Further inquiries can be directed to the corresponding author.
